# Biomechanical effects of cranial closing wedge osteotomy on joint stability in normal canine stifles: an ex vivo study

**DOI:** 10.1186/s12917-024-03923-1

**Published:** 2024-02-24

**Authors:** Masakazu Shimada, Chenxu Huang, Satoshi Yamakawa, Hiromichi Fujie, Sawako Murakami, Nobuo Kanno, Yasushi Hara

**Affiliations:** 1 Division of Veterinary Surgery, Department of Veterinary Science, Faculty of Veterinary Medicine, Nippon Veterinary and Life Science University, 1-7-1 Kyonan-cho, Musashino, Tokyo, 180-8602 Japan; 2 The Biomechanics Laboratory, Faculty of System Design, Tokyo Metropolitan University, Minamiosawa, Hachioji- shi, Tokyo, Japan; 3 Department of Sports Medical Biomechanics, Graduate School of Medicine, Osaka University, Suita, Osaka Japan

**Keywords:** Biomechanics, Cranial closing wedge osteotomy, Cranial cruciate ligament, Dog, Stifle joint, Six-degree-of-freedom robotic joint biomechanical testing system

## Abstract

**Background:**

Cranial closing wedge osteotomy (CCWO) is a functional stabilisation technique for cranial cruciate ligament (CrCL) ruptures. This biomechanical study aimed to evaluate the influence of CCWO on the stability of the stifle joint.

Eighteen Beagle stifle joints were divided into two groups: control and CCWO. The stifle joints were analyzed using a six-degree-of-freedom robotic joint biomechanical testing system. The joints were subjected to 30 N in the craniocaudal (CrCd) drawer and proximal compression tests and 1 Nm in the internal–external (IE) rotation test. Each test was performed with an extension position, 135°, and 120° of joint angle.

**Results:**

The stifle joints were tested while the CrCLs were intact and then transected.

In the drawer test, the CCWO procedure, CrCL transection, and stifle joint flexion increased CrCd displacement. The CCWO procedure and CrCL transection showed an interaction effect. In the compression test, the CCWO procedure decreased and CrCL transection and stifle joint flexion increased displacement. In the IE rotation test, CCWO, CrCL transection, and stifle joint flexion increased the range of motion.

**Conclusions:**

CCWO was expected to provide stability against compressive force but does not contribute to stability in the drawer or rotational tests. In the CCWO-treated stifle joint, instability during the drawer test worsened with CrCL transection. In other words, performing the CCWO procedure when the CrCL function is present is desirable for stabilizing the stifle joint.

## Background

Cranial cruciate ligament (CrCL) rupture is a common cause of hindlimb lameness in dogs. The CrCL plays a vital role in maintaining stifle joint stability by preventing cranial tibial drawer movement, hyperextension of the stifle joint, and excessive internal tibial rotation [1–3]. In the stance phase, cranial tibial thrust (CrTT) is generated at the femorotibial joint, and the tibia is displaced cranially when the CrCL is ruptured [2–4]. 

Tibial plateau levelling osteotomy (TPLO) and cranial closing wedge osteotomy (CCWO) are commonly used techniques to alter the tibial plateau angle (TPA) and neutralise the CrTT for functional stabilization [5, 6] Slocum and Devine described CCWO in 1984, [7] and Slocum and Slocum described TPLO in 1993 [8]. TPLO showed stability under weight-bearing and facilitated quick recovery, regardless of body size [5, 9]. CCWO is still widely used when an appropriate TPLO saw blade cannot be selected for small dogs or when complications are feared with TPLO, such as when the TPA is excessively high [5, 10]. CCWO and TPLO reportedly have similar clinical outcomes [11, 12]. However, postoperative differences in gait between TPLO and CCWO have been reported [13]. Though biomechanical studies are important to understand these differences, CCWO has limited research compared to TPLO, for which many biomechanical studies have been conducted [3, 14, 15]. 

To understand the biomechanical properties of CCWO, we performed three tests. The Craniocaudal (CrCd) drawer test simulates the method to detect the cranial drawer sign [16]. The proximal compression test simulates the method to detect positive CrTT [16]. The internal–external (IE) rotational test mimics the method to check the laxity of rotation. These methods have been commonly used for clinical diagnosis. In a previous study, TPLO in the stifle joint without the CrCL was shown to be effective in contributing to stability in the cranial direction during compression instead of creating instability in the CrCd drawer and IE rotational tests [3]. We hypothesized that CCWO would yield similar results.

## Methods

### Specimen preparation

The stifle joints used in this study were obtained from 18 left stifles of healthy beagle dogs euthanized for reasons unrelated to this study, such as for non-orthopaedic research and surgical training approved by our university’s Animal Experiment Committee and Bioethics Committee (approval number: 2019 J-29, 30 K-9, 2020 S-50). Beagle dogs were commonly used in experiments and practical training in the country where this study was performed; other breeds were unavailable in our institution. The dogs were euthanized by an overdose of pentobarbital (100 mg/kg, IV) in unrelated research or training. All dogs were confirmed to have had cardiac arrest by ECG monitoring and auscultation and respiratory arrest, each for at least 5 min; then the corneal reflex was performed to certify death. Also, all dogs were obtained commercially (Oriental Yeast Co., Ltd., Tokyo, Japan.) for unrelated research or training.

The dogs were randomly divided into two groups without knowing their individual information (sex, weight, age, TPA, etc.): those that did not undergo CCWO (control stifles) and those that did (CCWO stifles). The stifle joints of each group were tested as intact joints; then the CrCLs were transected in the joints (CrCLT) to provide four test situations: Control-Intact, Control-CrCLT, CCWO-Intact, and CCWO-CrCLT.

The specimen was prepared according to a previously reported method [3]. All soft tissues, except the collateral ligaments, the cruciate ligaments, the menisci, the joint capsule, the patella, and the patellar ligament, were removed from the stifle joint to create a bone-ligament-bone model. A mediolateral radiograph of each stifle joint was obtained, and the TPA was measured as described by Warzee et al. [15].

**Fig. 1:**
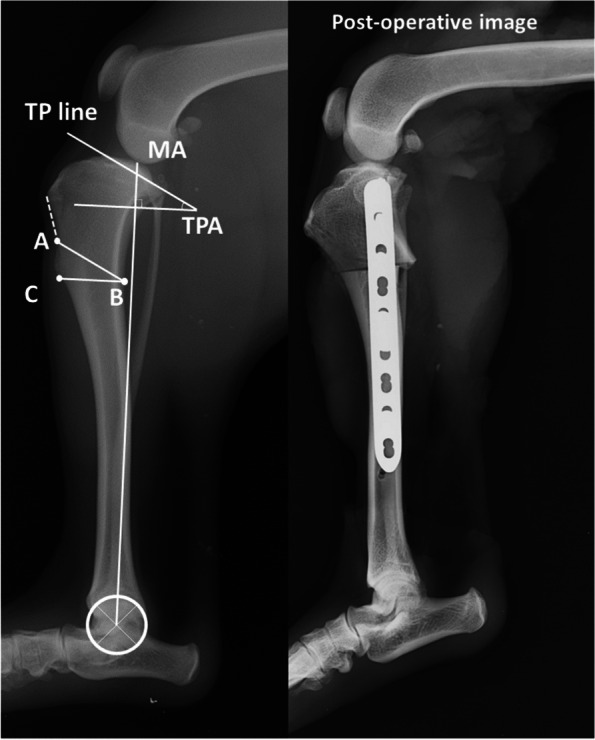
Preoperative planning of cranial closing wedge osteotomy. The tibial plateau angle (TPA) was determined from the tibial plateau line and the perpendicular line of mechanical axis of the tibia. Point A was on the cranial cortex and 10% of the tibial long axial length distally from the insertion of the patellar ligament. After determining point A, the proximal osteotomy line, parallel to the tibial plateau line and passing through A, was established. Point B was defined as the intersection of the proximal osteotomy line and the caudal cortex. A distal osteotomy line passing through B was drawn to make the angle with line AB equal to the TPA. Point C was defined as the intersection of the distal osteotomy line and the cranial cortex

The CCWO was planned based on preoperative radiographs (Fig. 1). Point A was on the cranial cortex and 10% of the tibial long axial length distally from the insertion of the patellar ligament. The CCWO technique has a lot of variability in osteotomy position as per previous reports [7, 10–12, 14]. Therefore, to minimise the variation in osteotomy position according to bone size, we standardized that position to 10% of the bone length and performed the procedure in this study. After determining point A, the proximal osteotomy line, parallel to the tibial plateau line and passing through A, was established. Point B was defined as the intersection of the proximal osteotomy line and the caudal cortex. A distal osteotomy line passing through B was drawn to make the angle with line AB equal to the TPA. Point C was defined as the intersection of the distal osteotomy line and the cranial cortex. Osteotomy was performed according to the plan, and bone segments were fixed with a 2.7-mm locking compression plate (Johnson & Johnson, New Brunswick, NJ.) so that the cranial aspects (points A and C) were aligned. After processing, radiographs were obtained again, and the postoperative TPA was measured (Fig. 1). The proximal femur and distal tibia were then fixed in cylindrical paper tubes. The bone was fixed with resin (GC Ostron II, GC Corporation, Tokyo, Japan.) so that the bony axis was positioned at the centre of the tube. The specimens were wrapped in gauze, soaked in lactated Ringer’s solution, and cryopreserved at -20 °C. They were thawed at 4 °C the day before testing.

### Testing protocol

**Fig. 2:**
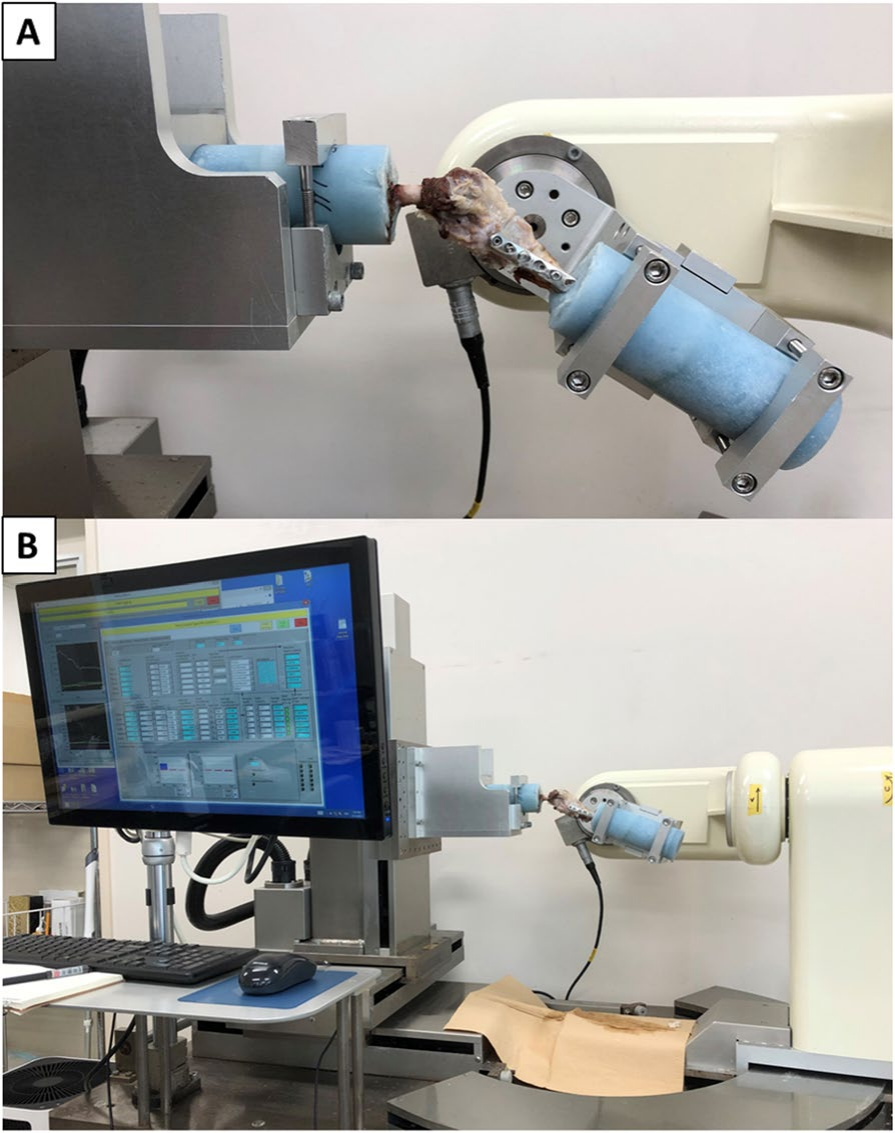
Robotic system. The testing system used in this study consists of a six-degree-of-freedom (6 DOF) manipulator with a 6 DOF universal force/moment sensor. Photograph of stifle joint installed in a robotic system. The femoral side of the robot controls the three translations (medial–lateral, cranial–caudal, proximal–distal), and the tibial side of the robot controls the three rotational movements (flexion–extension, internal–external, varus–valgus)

A custom-made six-degree-of-freedom (6 DOF) robotic testing system developed by Fujie et al. was used for testing (Fig. 2) [17–20]. This system enables the simulation of physiological stifle joint motion controlled with respect to either position or force using a coordinated ex vivo system. The coordinate system consists of three axes used to assess the rotation and translation in 6 DOF [3, 21]. The motion can be defined in terms of three rotations (flexion–extension [FE], IE, varus–valgus [VV]) and three translations (medial–lateral [ML], CrCd, proximal–distal [PD]). In the robotic system, the FE axis was defined using the insertion of the medial and the lateral collateral ligaments of the femur, and the IE rotation axis was defined as the longitudinal axis of the femur. The VV axis was the line perpendicular to the FE and IE rotation axes.

The caudal angle between the longitudinal axis of the femur and tibia was used to determine the tested angle. However, different methods were used to determine the extended position. For the control group, after the stifle joint was fixed to the system, extension torque was applied up to 0.7 Nm to the joint while keeping the other five DOFs at 0 N (CrCd, PD, and ML) and 0 Nm (VV and IE) using force control to extend the joint. This state was defined as the ‘extension position’ for the control group, and the joint angle was determined. The torque to be applied was determined from preliminary experiments performed on two stifle joint specimens before this study. When the extension torque was applied up to 1 Nm, the displacement decreased at approximately 0.7 Nm, and the stress–displacement curve was similar to that of the plateau. In a previous study [3] using the same robotic system, the mean angle at the maximum extension position of the normal stifle joint was 153°; therefore, this was used as the extension angle for the CCWO group. The CrCd drawer, proximal compression, and IE rotation tests were performed under the stifle joints at the extension position, 135°, and 120°, respectively.

In the CrCd drawer test, 30 N of CrCd force was applied to the tibia while maintaining the joint angle and keeping the other four DOF forces/torques at 0. The 6 DOF displacement was recorded during the test, and the CrCd displacement on the tibia relative to the femur was calculated. In the proximal compression test, 30 N of proximal force was applied to the tibia along its longitudinal axis while maintaining the FE and IE rotation angles and keeping the ML, VV, and CrCd forces at 0; the 6 DOF displacement was then recorded during the test, and the CrCd displacement on the tibia relative to the femur was calculated. In the IE rotation test, an IE torque of 1 Nm was applied to the tibia while maintaining the joint angle and the other four DOF forces/torques at 0. The 6 DOF displacement was then recorded, and the range of motion during IE rotation was calculated.

The stifle joints of each group were tested as intact joints; subsequently, the CrCLs were transected in the joints (CrCLT) to provide four test situations.

### Statistical analysis

SAS software (SAS software Ver 9.3; SAS Institute Inc., Cary, NC) was used for the statistical analysis. The skewness–kurtosis test was used to confirm a normal distribution. Comparisons of age, body weight, and preoperative TPA between groups (control stifles vs. CCWO stifles) were conducted using the Student’s t-test.

Statistical analyses of the values from each test were conducted by repeated-measures analysis of variance (ANOVA) using the Proc Mixed procedure of the SAS software. The linear model included the fixed effects of each group, joint angle, CrCL presence or absence, and their interactions. Each stifle joint was included as a random repeated effect. Multiple comparisons of the estimated least squares mean of the four situations were performed using the Tukey–Kramer test. The significant level was set at 0.05. In the Results, the interaction between each group and joint angle is shown as CCWO × Angle, the interaction between each group and CrCL presence or absence as CCWO × CrCL, and the interaction between CrCL presence or absence and joint angle as CrCL × Angle.

## Results

### Animals

No orthopaedic disease was found in any of the dogs. No radiographic findings suggestive of osteoarthritis, such as osteophyte or fat pad signs, were observed in any stifle joints. Macroscopic findings showed that the joints had no ligament damage. The study included seven females (age, 22.9 ± 7.1 months; body weight, 10.1 ± 0.6 kg) and 11 males (age, 19.5 ± 4.5 months; body weight, 11.4 ± 1.1 kg). The control group included stifle joints from nine dogs (seven males and two females; age, 21.9 ± 5.7 months; body weight, 11.3 ± 1.2 kg), and the CCWO group included stifle joints from nine dogs (four males and five females; age, 19.7 ± 6.0 months; body weight, 10.5 ± 0.9 kg). There were no statistically significant differences in age or body weight between the groups (age: *p* = 0.928, body weight: *p* = 0.133). The TPA was 31.7°±2.5° in the control group and 30.1°±1.9° in the CCWO group before surgery (preoperative TPA: *p* = 0.052). TPA was 6.6°±3.0° after the operation in the CCWO group.

### CrCd displacement on the tibia relative to the femur in the CrCd drawer test

**Fig. 3:**
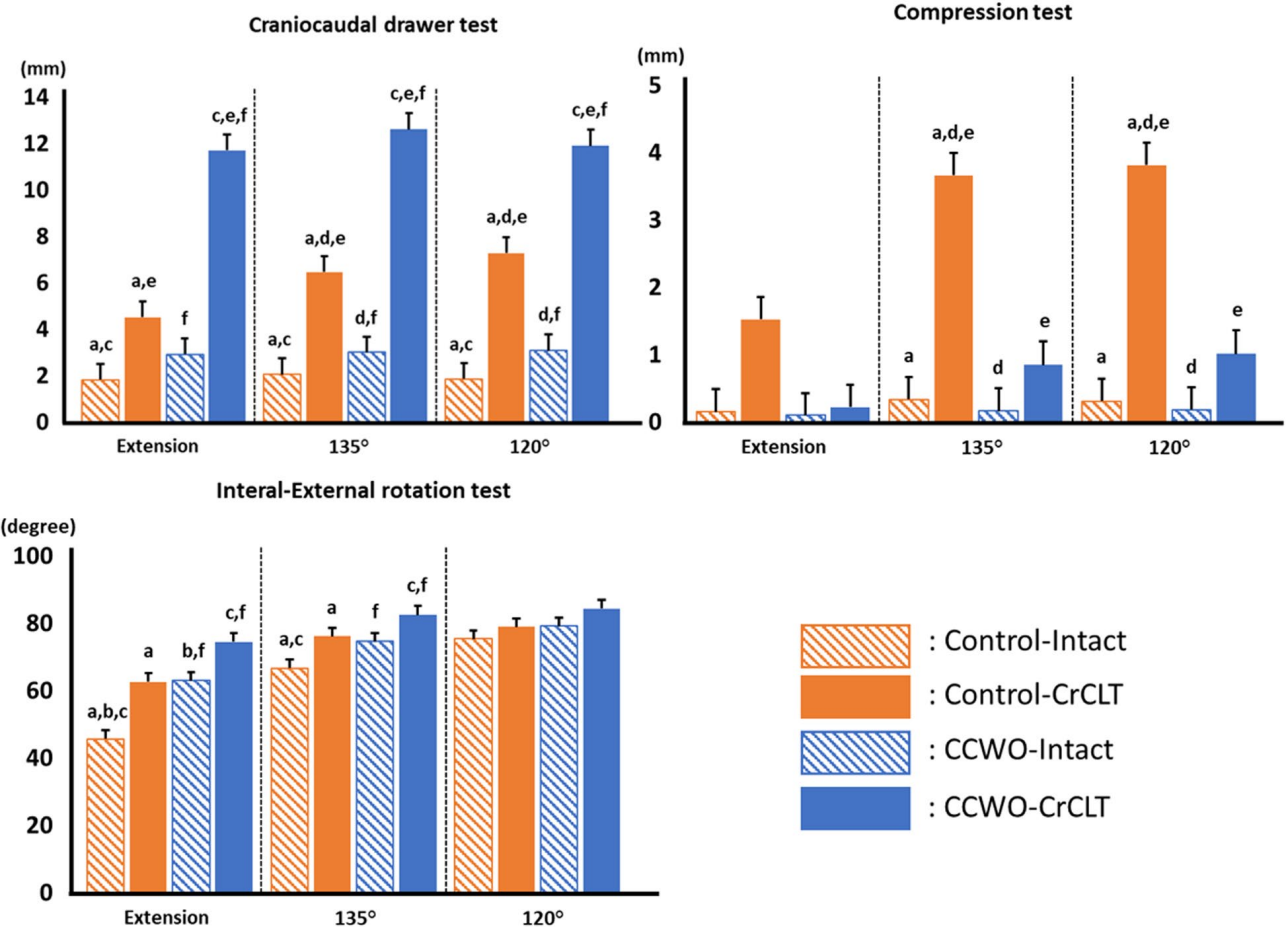
Result of each test. Data are presented as least square means and standard deviations. The striped orange bars show the Control-Intact, the solid orange bars show the Control-cranial cruciate ligament-transected (CrCLT), the striped blue bars show the cranial closing wedge osteotomy (CCWO)-Intact, and the solid blue bars show the CCWO-CrCLT. The x-axis is the joint angle of each group. The y-axis is craniocaudal displacement (mm) at the craniocaudal drawer and compression tests and internal-external range of motion (degree) at the internal–external axial rotation test. ^a^ *p* < 0.05, Control-Intact vs. Control-CrCLT; ^b^ *p* < 0.05, Control-Intact vs. CCWO-Intact; ^c^ *p* < 0.05, Control-Intact vs. CCWO-CrCLT; ^d^ *p* < 0.05, Control-CrCLT vs. CCWO-Intact; **e** *p* < 0.05, Control-CrCLT vs. CCWO-CrCLT; ^f^ *p* < 0.05, CCWO-Intact vs. CCWO-CrCLT

The CCWO procedure, CrCL transection, and stifle joint flexion increased displacement (ANOVA: CCWO, *p* < 0.001; CrCL, *p* < 0.001; Angle, *p* = 0.045). In particular, the CCWO procedure and the CrCL transection synergistically increased the displacement (Interaction: CCWO × CrCL; *p* < 0.001) (Fig. 3; Tables 1 and 2).

**Table 1:** Comparison of the outcomes between the four test situations

	Craniocaudal displacement in the craniocaudal drawer test (mm)			
	Control-Intact	Control-CrCLT	CCWO-Intact	CCWO-CrCLT
Extension	1.8 ± 0.69^a,c^	4.3 ± 0.69^a,e^	2.9 ± 0.69^f^	10 ± 0.69^c,e,f^
135°	2.1 ± 0.69^a,c^	6.2 ± 0.69^a,d,e^	2.9 ± 0.69^d,f^	11 ± 0.71^c,e,f^
120°	1.9 ± 0.69^a,c^	6.9 ± 0.69^a,d,e^	2.8 ± 0.69^d,f^	12 ± 0.71^c,e,f^
	**Craniocaudal displacement in the proximal compression test (mm)**			
	Control-Intact	Control-CrCLT	CCWO-Intact	CCWO-CrCLT
Extension	0.15 ± 0.33	1.5 ± 0.33	0.097 ± 0.33	0.22 ± 0.33
135°	0.33 ± 0.33^a^	3.7 ± 0.33^a,d,e^	0.16 ± 0.33^d^	0.85 ± 0.35^e^
120°	0.31 ± 0.33^a^	3.8 ± 0.33^a,d,e^	0.18 ± 0.33^d^	1.0 ± 0.35^e^
	**Internal–external range of motion in the internal–external axial rotation test (°)**			
	Control-Intact	Control-CrCLT	CCWO-Intact	CCWO-CrCLT
Extension	46 ± 2.5^a,b,c^	63 ± 2.5^a^	63 ± 2.5^b,f^	75 ± 2.5^c,f^
135°	67 ± 2.5^a,c^	76 ± 2.5^a^	75 ± 2.5^f^	83 ± 2.6^c,f^
120°	75 ± 2.5	79 ± 2.5	79 ± 2.5	84 ± 2.6

Data are presented as least square means and standard deviations

*CCWO* Cranial closing wedge ostectomy, *CrCLT* Cranial cruciate ligament-transected

^a^ *p* < 0.05, Control-Intact vs. Control-CrCLT

^b^ *p* < 0.05, Control-Intact vs. CCWO-Intact

^c^ *p* < 0.05, Control-Intact vs. CCWO-CrCLT

^d^ *p* < 0.05, Control-CrCLT vs. CCWO-Intact

^e^ *p* < 0.05, Control-CrCLT vs. CCWO-CrCLT

^f^ *p* < 0.05, CCWO-Intact vs. CCWO-CrCLT

**Table 2:**
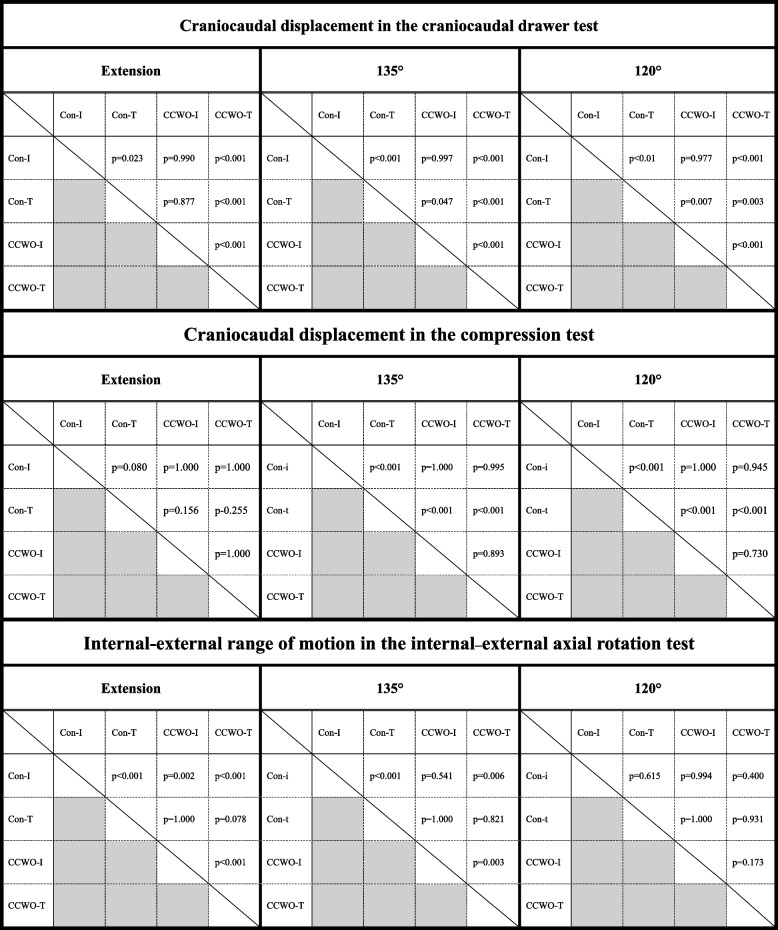
*P* values for comparison between the four situations

*Con* Control, *CCWO* Cranial closing wedge osteotomy, *I* Intact, *T* Cranial cruciate ligament-transected

### CrCd displacement on the tibia relative to the femur in the compression test

The CCWO procedure decreased displacement, and CrCL transection and stifle joint flexion increased displacement (ANOVA: CCWO, *p* < 0.001; CrCL, *p* < 0.001; Angle, *p* = 0.001). In addition, the CCWO procedure offsettingly reduced displacement relative to the CrCL transection. Also, the CrCL transection and the joint flexion synergistically increased the displacement (Interaction: CCWO × CrCL; *p* < 0.001, CrCL × Angle; *p* = 0.003) (Fig. 3; Tables 1 and 2).

### IE range of motion on the tibia relative to the femur at the IE rotational test

The CCWO procedure, CrCL transection, and stifle joint flexion increased the range of motion (ANOVA: CCWO, *p* = 0.02; CrCL, *p* < 0.001; Angle, *p* < 0.001). In addition, the CCWO procedure and the joint flexion synergistically increased the displacement and so did the CrCL transection and the joint flexion (Interaction: CCWO × Angle; *p* < 0.001, CrCL × Angle; *p* = 0.003) (Fig. 3; Tables 1 and 2).

## Discussion

The results of this study indicated that CCWO could be expected to provide stability against CrTT under compression force but does not contribute to CrCd stability under the drawer test or IE rotational stability under the IE test. In addition, especially in the CCWO-treated stifle joint, instability during the drawer test worsened with CrCL transection.

In this study, the TPA after CCWO was 6.6°±3.0°, and stability for compression was achieved. The previously reported ex vivo study on CCWO stability focused on cranial subluxation of the tibia during compression loading [14]. The study reported that correcting TPA to 4°–6° could neutralise subluxation during compression loading [14]. In a study of TPLO using the same robotic system, the TPA was changed to 6.4°, and stability under compression tests was obtained [3]. In other words, it was shown that stability could be obtained for CCWO with the TPA similar to TPLO.

In this study, CCWO does not contribute to stability in the CrCd drawer or IE rotational tests. Particularly in the CCWO-treated stifle joint, instability during the drawer test worsened with CrCL transection. Because the collateral ligaments relax with flexion, [22] such flexion of the articular surface risks contributing to instability in TPLO [3, 23]. The CCWO may also change the joint surface into relative flexion. In the presence of the CrCL, stability in the CrCd drawer test was not affected by CCWO. For cranial loading, the CrCL has a strong effect on stability, regardless of the joint angle [24]. However, with the transection of the CrCL, stability in the cranial direction was lost. CCWO and transection of the CrCL had a synergistic effect, contributing to instability in the craniocaudal direction. The IE test also showed that CCWO contributed to instability in the stifle joint during extension. This result was similar to a previous study, where TPLO had a significant effect on rotational stability [3]. CCWO may contribute to the instability of CrCd drawer movement and IE rotation without compressive loads.

The clinical outcomes of TPLO and CCWO, such as owner satisfaction and subjective postoperative gait evaluation, are similar [11, 12]. This may be explained by the fact that CCWO, similar to TPLO, stabilises CrTT in the stance phase. Recently, much attention has been paid to the early adaptation of TPLO, which may slow osteoarthritis that occurs after partial tears of the CrCL [25–27]. It is possible that the residual function of the CrCL minimises the instability associated with TPLO during non-weight bearing [3]. Considering that CCWO also had minimal instability when the CrCL was intact in this study, early adaptation of CCWO is expected to slow the progression of osteoarthritis. A previous study reported that the CCWO procedure was more likely to result in hyperextended stifle gait patterns of the swing phase postoperatively than the TPLO procedure in kinematics analysis [13]. Our study focused on static stabilisers and suggested that CCWO may not differ from TPLO, indicating dynamic stabilisers impact these gait changes. For example, it has been noted that CCWO causes downward traction of the patella due to the inclusion of the tibial tuberosity, the attachment site of the quadriceps muscle, in the proximal fragment [28, 29]. Therefore, future studies on the effects on dynamic stabilisers would provide a better understanding of CCWO.

This study had several limitations. First, it used healthy dogs without cranial cruciate ligament disease (CCLD), which may yield different results from clinical cases, as periarticular fibrosis may contribute to some degree of stability in clinical CCLD cases. Second, this study was unable to compare the control and CCWO using the same specimens. Therefore, each group differed according to sex. The effect of sex on the stability of the stifle joint is unknown, as previous reports did not indicate the effect of sex on the biomechanical properties of dogs. A recent review of etiopathogenetic factors reported that the main predisposing factors for rupture of the CrCL include age between 2 and 10 years, having been neutered or spayed, and being large and/or overweight [30]. Notably, the population in this study was at little risk of having these predisposing factors. The small sample size was also a notable limitation, as there was a limit to the number of specimens that could be used. Since the analysis was performed on a single breed, the beagle, there is a risk that the results may differ depending on the anatomical morphology associated with different breeds. Depending on the studies, each fragment has variations in the osteotomy line and position. Differences in the osteotomy position and the fixation position of the proximal and distal bone fragments are factors that modify the functional tibial axis [31]. In this study, to unify the conditions of the osteotomy, the osteotomy position was determined relative to the size of the tibia and the proximal and distal fragments fixed with the cranial cortices aligned. Therefore, it is conceivable that the CCWO using previous methods may provide different results.

## Conclusion

Although CCWO can contribute to stability against CrTT under compressive loading, the CrCd drawer test revealed a risk of the joint becoming more unstable, particularly in the absence of CrCL. Therefore, applying CCWO before the loss of function of the CrCL may minimise stifle joint instability and slow the progression of osteoarthritis. Future work is expected to clarify the differences between CCWO and TPLO in vivo, including the effects on dynamic stabilisers.

## Data Availability

The data supporting the findings of this study are available from the corresponding author upon reasonable request.

## Abbreviations

CCWO: Cranial closing wedge osteotomy
CrCL: Cranial cruciate ligament
CrCd: Craniocaudal
IE: Internal-external
CrTT: Cranial tibial thrust
CrCLT: Cranial cruciate ligament-transected
TPLO: Tibial plateau levelling osteotomy
TBA: Tibial plateau angle
FE: Flexion–extension
VV: Varus–valgus
ML: Medial–lateral
PD: Proximal–distal
DOF: Degree-of-freedom
CCLD: Cranial cruciate ligament disease
